# Compositional difference in antioxidant and antibacterial activity of all parts of the *Carica papaya* using different solvents

**DOI:** 10.1186/s13065-016-0149-0

**Published:** 2016-02-03

**Authors:** Nazia Asghar, Syed Ali Raza Naqvi, Zaib Hussain, Nasir Rasool, Zulfiqar Ali Khan, Sohail Anjum Shahzad, Tauqir A. Sherazi, Muhammad Ramzan Saeed Ashraf Janjua, Saeed Ahmad Nagra, Muhammad Zia-Ul-Haq, Hawa Ze Jaafar

**Affiliations:** Department of Chemistry, Government College University, Faisalabad, 38000 Pakistan; Institute of Chemistry, University of the Punjab, Lahore, 54000 Pakistan; Department of Chemistry, COMSATS Institute of Information Technology, Abbottabad, 22060 Pakistan; Department of Chemistry, University of Sargodha, Sargodha, 40100 Pakistan; The Patent Office, Karachi, Pakistan; Department of Crop Science, Faculty of Agriculture, UPM, 43400 Serdang, Selangor Malaysia

## Abstract

**Background:**

*Carica papaya* is a well known medicinal plant used in the West and Asian countries to cope several diseases. Patients were advised to eat papaya fruit frequently during dengue fever epidemic in Pakistan by physicians. This study was conducted to establish Polyphenols, flavonoids and antioxidant potential profile of extracts of all major parts of the *C. papaya* with seven major solvents i.e. water, ethanol, methanol, n-butanol, dichloromethane, ethyl acetate, and n-hexane.

**Results:**

TPC, TFC, antioxidant and antibacterial potential were determined using different aqueous and organic solvents in addition to the determination of trace element in leaves, pulp and peel of *C. papaya*. Total soluble phenolics and flavonoids were found in promising quantity (≈66 mg GAE/g) especially in case of methanol and ethanol extracts. Antioxidant activity using DPPH free radical scavenging assay indicated leaves, bark, roots and pulp extracts showed >75.0 % scavenging potential while leaves and pulp showed 84.9 and 80.9 % inhibition of peroxidation, respectively. Reducing power assay showed leaves, pulp and roots extracts active to reduce Fe^3+^ to Fe^2+^ ions. The antibacterial study showed pulp extract is the best to cope infectious action of bacteria.

**Conclusion:**

This study was conducted to test the medicinal profile of all parts of *C. papaya* by extracting secondary metabolites with organic and aqueous solvents. Ethanol and methanol both were found to be the best solvents of choice to extract natural products to get maximum medicinal benefits and could be used to medicinal formulation against different infectious diseases.

**Graphical abstract:**
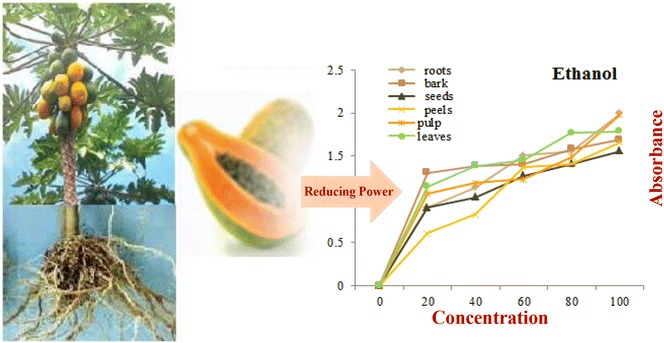
Medicinal evaluation of different parts of C. papaya.

## Background

It is no doubt a common person knows the nutritional values of the vegetables and fruits in sense of maintaining the health and preventing the diseases because of vitamins and some special compounds. Yes; they are true in their claim because they don’t know about what these compounds perform in their body to make them healthy. Most of the compounds present in fruits and vegetables may modify a multitude of mechanisms that are known in proliferation of diseases. The rest of the nutrients may take part in body building. However, it is widely accepted that these are the fruits and vegetables that have potential to reduce the risk of oxidative stress related diseases [1]. Recent studies have investigated the role of dietary factors in reducing the risk of chronic disease. The results of these investigations concluded if a person who set the fruits and vegetables a necessary part of his diet could reduce >50 % the risk of oxidative stress diseases and cancer particularly gastrointestinal tract cancer. Understanding of the relationship between food nutrients and health is very necessary as there are about 25,000 biologically active compounds which have ability to cope with oxidants working in human body directly or indirectly [2–4].

Oxidants mainly the free radical moieties such as nitric monoxide (NO^·^), superoxide (O_2_^−)^ and hydroxyl (OH^·^) and molecules like hydrogen peroxide (H_2_O_2_) and peroxynitrite (ONOO^−^) are produced as a result of numerous physiological and biochemical processes. Although these species perform key biological functions in body such as oxygen carrier radicals involve in regulation of soluble guanylatecyclase activity, signal transduction and gene transcription; nitrogen carrier species involve in leukocytes adhesion, hemodynamics, thrombosis, platelets aggregation, signaling molecule that essentially regulate the relaxation and proliferation of vascular smooth muscle cells, angiogenesis and vascular tone [5]. In addition to these activities, these moieties also involve in oxidative damage to lipid, proteins and DNA in living bodies that cause many chronic diseases e.g. cancer, cardiovascular, diabetics etc. ROS play crucial role in growing the chronic disorders because it attacks especially free radical sensitive cells such as post-mitotic glial cells and neurons which lead to cardiovascular, neurodegenerative diseases and cancer [6].

All these species which have serious deleterious effect in human body no longer free in the presence of antioxidants to perform its damaging action in body. Antioxidants are those species which deplete or at least debilitate the function of the oxidants. At first our body itself produces some compounds known as endogenous compounds in response to the free radicals or oxidants generation to fix its action. However, overproduction of the free radicals or ROS or oxidants in body suppresses or even deactivates the endogenous antioxidant defensive system. Over production of free radicals might be due to the extensive electromagnetic radiation exposure, eating non-food grade dietary items, and extensive muscular work. Unchecked over production of free radicals may cause highly chronic diseases such as aging, Parkinson’s disease, Alzheimer’s disease and many other neural disorders. These disorders could be slow down or even cured using exogenous compounds (natural or synthetic) [7–9]. Natural antioxidants are enzymatic or non-enzymatic moieties. Polyphenols, carotenoids are famous non-enzymatic antioxidants which are mainly present in nuts, vegetables and fruits. Regular intake of vegetables and fruits dramatically reduce the oxidative stress and its allied risks. Antioxidant components of the fruits and vegetables are responsible for scavenging of free radicals, RNS, ROS, and inhibiting the process trigger the ROS generation [10].

*Carica papaya* fruit which belongs to the family *Caricaceae* grown in different areas of the world, is one of them which are well recognized as a potential medicinal fruit possessing unique food values and biological potentials [11]. Medicinal uses of different parts of *C. papaya* has been reported such as leaves smoke were used for asthma relief and poultice for nervous pains, pulp for preventing rheumatism and urine acidity, and flowers for jaundice and hypertension [12, 13]; however medicinal uses of *C. papaya* vary from area to area. In Pakistan it is suggested by physicians to dengue fever patients to eat papaya in good quantity due to its immune booster, antiviral and antioxidant properties. In this study we determined the antioxidant and antibacterial potential profile of all major parts extracts of the *C. papaya* in seven common organic and aqueous solvents.

## Results and discussion

### Extraction yield

The results showed that the extraction yields obtained was affected by the solvent used as shown in Table 1. Difference in yields of extracts affected with polarity of solvents and various compounds present in different parts of the *C. papaya*. The highest yield was obtained by the aqueous solvent; 29/100 g dry powder of roots and 28/100 g dry powder of leaves. The poorest yield was achieved with n-hexane (0.4/100 g dry powder of pulp). The extraction yield was obtained in the following descending order; water>methanol>ethanol>ethyl acetate>dichloromethane>n-butanol>n-hexane. Polarity of the solvent, nature of the extracted compounds and extraction process highly affects antioxidant and antibacterial activities of the plant extracts [14].

**Table 1:** Extraction yield (g/100 g dry matter) of different parts of *C. papaya* in seven different solvents (mean; *n* = 3)

Extracting Solvents	Roots	Bark	Peels	Pulp	Seeds	Leaves
n-Hexane	03.95	05.99	04.66	00.40	00.71	08.56
Dichloromethane	07.10	24.22	09.83	08.74	06.27	13.85
n-Butanol	10.10	09.09	04.15	02.70	01.32	08.25
Ethyl acetate	17.00	10.93	12.27	11.48	10.12	18.89
Water	29.00	21.92	22.00	14.57	12.84	28.00
Methanol	19.46	10.90	16.50	13.32	14.32	15.90
Ethanol	20.89	14.47	15.66	10.31	12.36	11.47

### Metal profile

Metals are present in earth’s crust and its contents distribute in the nature through food cycle and energy cycle. Trace metals are necessary entities of biological systems to trigger and regulate the key body functions. Fruits and vegetables are main sources of trace elements such as iron (Fe), zink (Zn), cobalt (Co) and copper (Cu) which combines with certain biomolecules to produce enzymes and co-enzymes to catalyze and trigger certain body functions [15, 16]. Trace elements also assist the endogenous antioxidant activities. Without processing pulp is the most common edible part of fruits to fulfill the nutritional requirement of trace elements. The results showed *C. papaya* pulp contain trace amount of Fe, and Zn (2.56 and 0.06 respectively) and very poor quantity of Cu. However, a good quantity was detected in leaves and peels as shown in Table 2. The routine use of peel and leaves is not possible as pulp but the extracts of leaves and peels could be used as mineral source after necessary processing in addition to antioxidants source.

**Table 2:** Metal profile of *C. papaya* leaves, pulp and peel

Sample	Iron (Fe)	Lead (Pb)	Cobalt (Co)	Copper (Cu)	Zinc (Zn)
Leaves	13.55 ± 0.01	5.00 ± 0.01	0.01 ± 0.00	7.75 ± 0.02	20.01 ± 0.00
Pulp	2.56 ± 0.01	ND	ND	0.00 ± 0.00	0.06 ± 0.00
Peels	0.88 ± 0.40	3.00 ± 0.1	0.02 ± 0.03	4.01 ± 0.90	10.03 ± 0.00

Mean ± S.E (ng/100 g dry extract)

*ND* not detected

### Determination of total phenolic contents

Nutritional values of food mainly based on TPC and TFC profile. Both contents are considered the index of medicinal values of natural products [17]. TPC was determined by standard method using Folin-Ciocalteu reagent and the results were expressed in term of mg GAE/g dry matter (Table 3). The organic solvent extracts of different parts of *C. papaya* had prominent yield. TPC determined in different parts of the *C. papaya* ranging from 1.22–65.12 mg GAE/g dry powder. The most extractable solvents of phenolics were the ethanol and methanol. The highest phenolic compounds was achieved with ethanol (65.12 mg GAE/g dry leave powder and 61.25 mg GAE/g dry bark powder) followed by methanol solvent (54.28 mg GAE/g dry leave powder). Whereas poorest phenolics were obtained with dichloromethan solvent (1.2 mg GAE/g dry root powder). Vuong et al. (2013) reported TPC of *C. papaya* fruit extracts with methanol and ethanol solvent 15.03 and 9.43 mg GAE/g dry powder, respectively. These contents were lower than we determined, however leave extract with ethanol solvent showed 63.59 mg GAE/g crude powder which is in good agreement with our results (65.12 mg GAE/g dry powder of leave) [17]. Other organic solvents such as n-hexane, n-butanol, and ethyl acetate showed mild extraction yield. The poor extraction could be explained on the bases that these solvents contain dominant non-polar nature character while methanol and ethanol both contain moderate polar to non-polar behavior which is more favorable to extract phenolics and flavonoids. Comparatively less extraction of phenolics with water solvent is due to the extraction with high percentage of impurities [18].

**Table 3:** Total phenolic contents (mg GAE/g) values of all major part extracts in aqueous and organic solvents of *C. papaya* (mean ± SE; *n* = 3)

Extracting Solvents	Leaves	Bark	roots	Peels	seeds	pulp	LSD 5 %
n-Hexane^***^	10.60 ± 0.06^b^	09.85 ± 0.03^c^	02.64 ± 0.00^f^	07.32 ± 0.05^d^	06.74 ± 0.02^e^	15.92 ± 0.03^a^	0.07
Dichloromethane^***^	11.77 ± 0.03^e^	21.60 ± 0.04^a^	01.22 ± 0.01^f^	21.15 ± 0.12^b^	16.02 ± 0.03^d^	19.62 ± 0.04^c^	0.10
n-Butanol^***^	21.69 ± 0.03^c^	25.80 ± 0.04^a^	05.83 ± 0.02^e^	24.80 ± 0.04^b^	25.85 ± 0.09^a^	20.93 ± 0.04^d^	0.09
Ethyl acetate^***^	27.80 ± 0.02^d^	28.80 ± 0.05^c^	09.39 ± 0.05^f^	27.21 ± 0.14^e^	32.52 ± 0.49^a^	31.88 ± 0.01^b^	0.37
Water^***^	49.94 ± 0.60^a^	31.31 ± 0.05^d^	19.92 ± 0.04^f^	32.23 ± 0.64^c^	27.94 ± 0.09^c^	37.78 ± 0.11^b^	0.65
Methanol^***^	54.28 ± 0.10^a^	37.09 ± 0.52^d^	41.72 ± 0.54^b^	35.15 ± 0.53^e^	38.86 ± 0.82^c^	38.15 ± 0.53^c^	0.89
Ethanol^***^	65.12 ± 1.21^a^	61.25 ± 0.10^b^	49.08 ± 0.09^c^	43.79 ± 1.20^e^	43.42 ± 0.06^f^	48.49 ± 0.18^d^	0.27

Values with same letter in superscript in row do not differ significantly

*NS* non-significant

*** Significant at 0.001 level

**Fig. 1:**
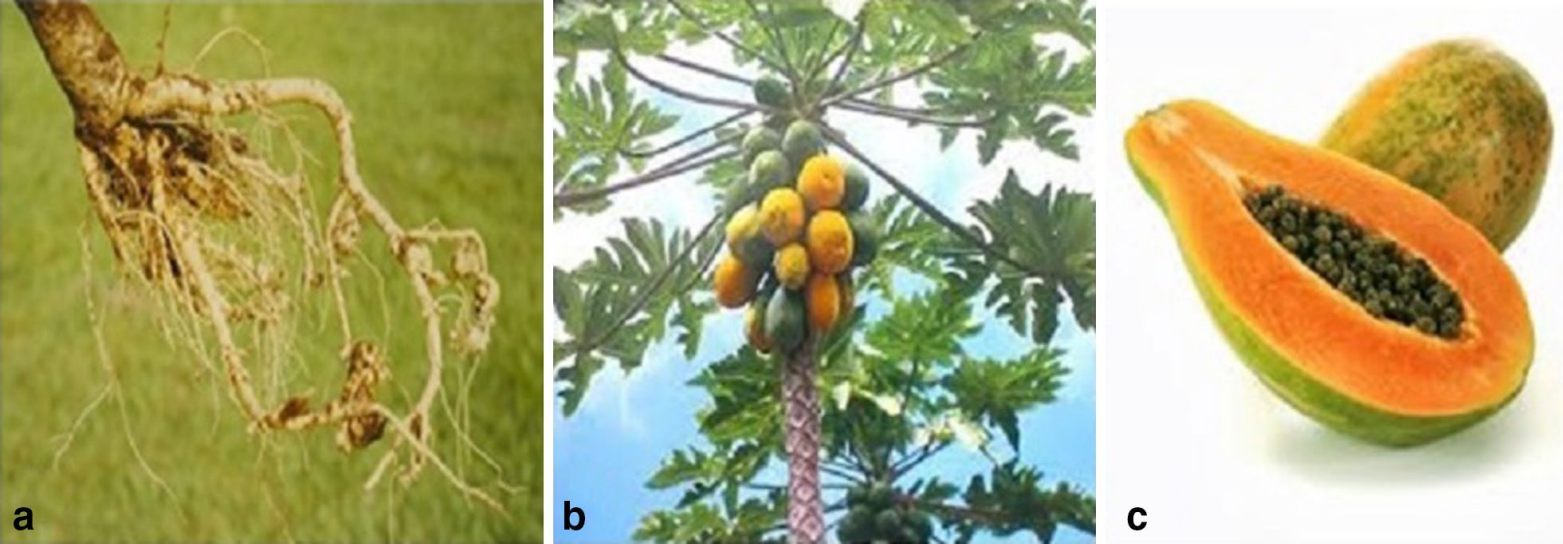
Major parts of *C. papaya* **a** roots **b** leaves, bark and fruit and **c** fruit pulp and seeds

All phenolic contents do not have equal antioxidant strength; it is investigated highly polar phenolic contents extracted with water showed week antioxidant potential while mild polar phenolic contents commonly extracted with high yield with ethanol and methanol solvents showed awesome antioxidant potential which have great credibility in contrast to the synthetic antioxidants [19]. Synthetic antioxidants in addition to quench oxidation process were also found to involve in toxicity such as genotoxicity and carcinogenicity which is the key reason of reviving the attention toward natural products in recent years [20]. This study also has showed good extraction with ethanol solvent and also promising antioxidant and antibacterial potential as compared to other tested solvents. Statistical analysis showed strong significant difference in total phenolics among different parts (P ≤ 0.001) Fig. 1.

### Determination of total flavonoid contents

Flavonoids are the second important figure of natural extracts to evaluate the medicinal importance of plants. It is sub class of polyphenols having benzo-γ-pyrone structure. In literature more than 6000 flavonoid compounds have been cited that was identified in plants. Many of which are present in fruits and vegetables. These compounds are responsible to protect plants from microbial and insects attack while in human body play defensive role as anti-inflammatory, anti-microbial, anti-cancer and anti-oxidant moieties [21–24]. Flavonoids extraction was found to be depend on the solvent used as shown in Table 4. Statistical analysis showed strong significant difference among flavonoid contents of different parts (P ≤ 0.001). The highest flavonoid contents were extracted with ethanol solvent (21.88 mg CE/g dry powder) followed by methanol. The lowest contents (0.13 mg CE/g dry powder) were found in dichloromethane extract followed by n-hexane and n-butanol extracts. Harnly and co-workers (2006) calculated and determined the flavonoid compounds (flavan-3-ols, anthocyanins, flavanones, flavones, and flavonols) in US based 31 fruitrs and found the most prominent medicinally important fruits such as blackberries and blueberries contain promising quantity of flavonoids, 202.5 and 79.9 mg CE/100 g fresh samples respectively [25]. Both blackberries and blueberries are best known for its antimicrobial and anti-oxidant activities and are being marketed in the form of processed extracts to improve mental function, reduce the risk of cancer, as anti-aging agent, and overall improvement in health. Different parts of *C. papaya* also showed promising quantity of these valued compounds which could be further processed as a ready to use source of flavonoids.

**Table 4:** TFC values (mg CE/g dry powder) of all major part extracts in aqueous and organic solvent of *C. papaya* (mean ± SE; *n* = 3)

Extracting solvents	Leaves	Bark	Roots	Peels	Seeds	Pulp	LSD 5 %
n-Hexane^***^	05.70 ± 0.01^a^	00.60 ± 0.00^e^	00.59 ± 0.06^e^	01.10 ± 0.01^c^	00.90 ± 0.13^d^	04.90 ± 0.01^b^	0.10
Dichloromethane^***^	06.64 ± 0.07^b^	01.58 ± 0.01^d^	00.13 ± 0.01^f^	01.23 ± 0.12^e^	01.65 ± 0.01^c^	08.74 ± 0.02^a^	0.10
n-Butanol^***^	08.39 ± 0.02^b^	02.61 ± 0.02^e^	03.62 ± 0.25^d^	03.72 ± 0.01^d^	04.66 ± 0.04^c^	10.87 ± 0.02^a^	0.19
Ethyl acetate^ns^	11.20 ± 0.07^a^	08.59 ± 0.03^a^	10.21 ± 0.03^a^	08.57 ± 0.01^a^	10.21 ± 0.01^a^	11.23 ± 0.01^a^	423.41
Water^***^	12.61 ± 0.50^a^	10.11 ± 0.53^c^	12.37 ± 0.03^a^	12.93 ± 0.29^a^	06.56 ± 0.14^d^	12.06 ± 0.20^ab^	0.59
Methanol^***^	15.54 ± 0.12^b^	15.84 ± 0.25^b^	16.69 ± 0.22^a^	13.92 ± 0.13^c^	08.62 ± 0.16^d^	08.24 ± 0.08^e^	0.30
Ethanol^***^	21.88 ± 0.06^a^	18.20 ± 0.53^d^	18.99 ± 0.02^c^	19.81 ± 0.02^b^	10.44 ± 0.17^f^	16.26 ± 0.20^e^	0.41

Values with same letter in superscript in row do not differ significantly

*NS* non-significant

*** Significant at 0.001 level

## Antioxidant activities

### Determination of DPPH free radical scavenging potential

**Fig. 2:**
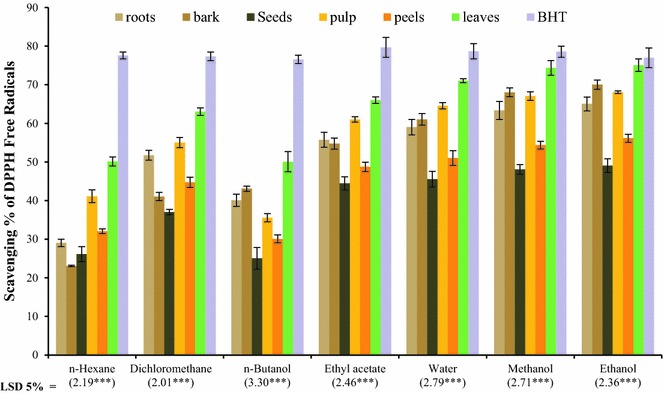
DPPH free radical scavenging activity study of all major part extracts in aqueous and organic solvents of *C. papaya* (mean ± SE; *n* = 3; *** = significant at 0.001 level)

Polyphenols are considered the index of antioxidant potential of fruits and vegetables. Different assays are being conducted to quantify the antioxidant strength. DPPH free radical scavenging assay is considered one of the best authentic assay for antioxidant study [26]. DPPH is an organic stable free radical which gives purple color in solution with maximum absorption at 517 nm (λ_max_) [27, 28]. On accepting an electron or free radical specie its color shifts from purple to yellow and also decrease in absorbance at λ_max_. This change in absorption makes the bases of anti-oxidant quantification. The DPPH free radical scavenging assay results showed significant difference in scavenging act ivity among different parts (P ≤ 0.01 and P ≤ 0.001) as shown in Fig. 2. It shows that the highest DPPH free radical scavenging potential was found with ethanol solvent extracts of leaves (75.05 %) followed by pulp extract with same solvent (68.07 %). *Carica papaya* bark and roots also showed promising DPPH radical scavenging potential; particularly in case of ethanol and methanol extracts in which bark extracts superseded the scavenging potential of pulp. The highest DPPH free radical scavenging potential of bark might be due to the promising quantity of phenolic and flavonoid contents in their extracts. The lowest DPPH free radical scavenging potential appeared in the case of n-hexane and n-butanol extracts (Fig. 2) that might be due to difference in polarity of extracted solvents and compounds.

### % Inhibition of linoleic acid peroxidation

**Fig. 3:**
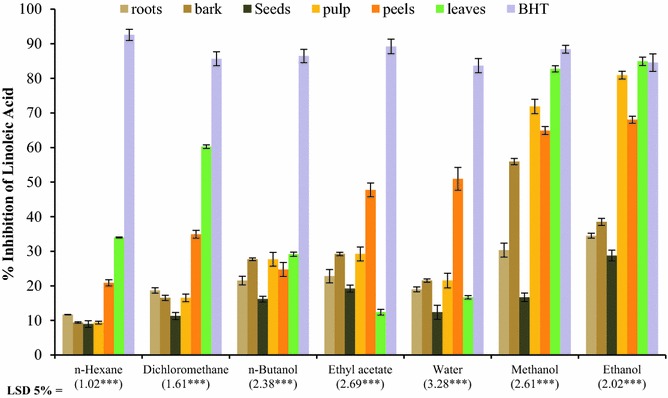
Percent inhibition of linoleic acid peroxidation study of all major part extracts in aqueous and organic solvents of *C. papaya* (mean ± SE; *n* = 3; *** = significant at 0.001 level)

Lipid peroxidation (oxidation of lipid) by ROS imposes deteriorated effect on human body and is a crucial step in the pathogenesis of several diseases. Generally ROS readily after its formation attacks the polyunsaturated fatty acids chain of cell membrane and start self-propagated chain reaction which ends in the damaging of cell and tissues and consequently the initiation of the disease. Fruits and vegetables with good potential to inhibit lipid peroxidation are considered most important. Percent inhibition of linoleic acid peroxidation by aqueous and organic solvents extracts of different parts of *C. papaya* showed strong significant difference (P ≤ 0.001) as shown in Fig. 3. The highest linoleic acid peroxidation inhibition was determined with ethanol solvent extract of leave which was 85 % followed by methanol extract (82 %) and ethanol extract of pulp (81 %). The lowest inhibition value was determined with n-hexane solvent extract of seeds (8 %). Other solvents (n-hexane, n-butanol, and dichloromethane) due to their mild polarity remained unable to extract antioxidants and consequently showed weak inhibition of linoleic acid peroxidation. Ethanolic extract superseded the BHT (control) potential to inhibit the linoleic acid peroxidation.

### Determination of reducing power

**Fig. 4:**
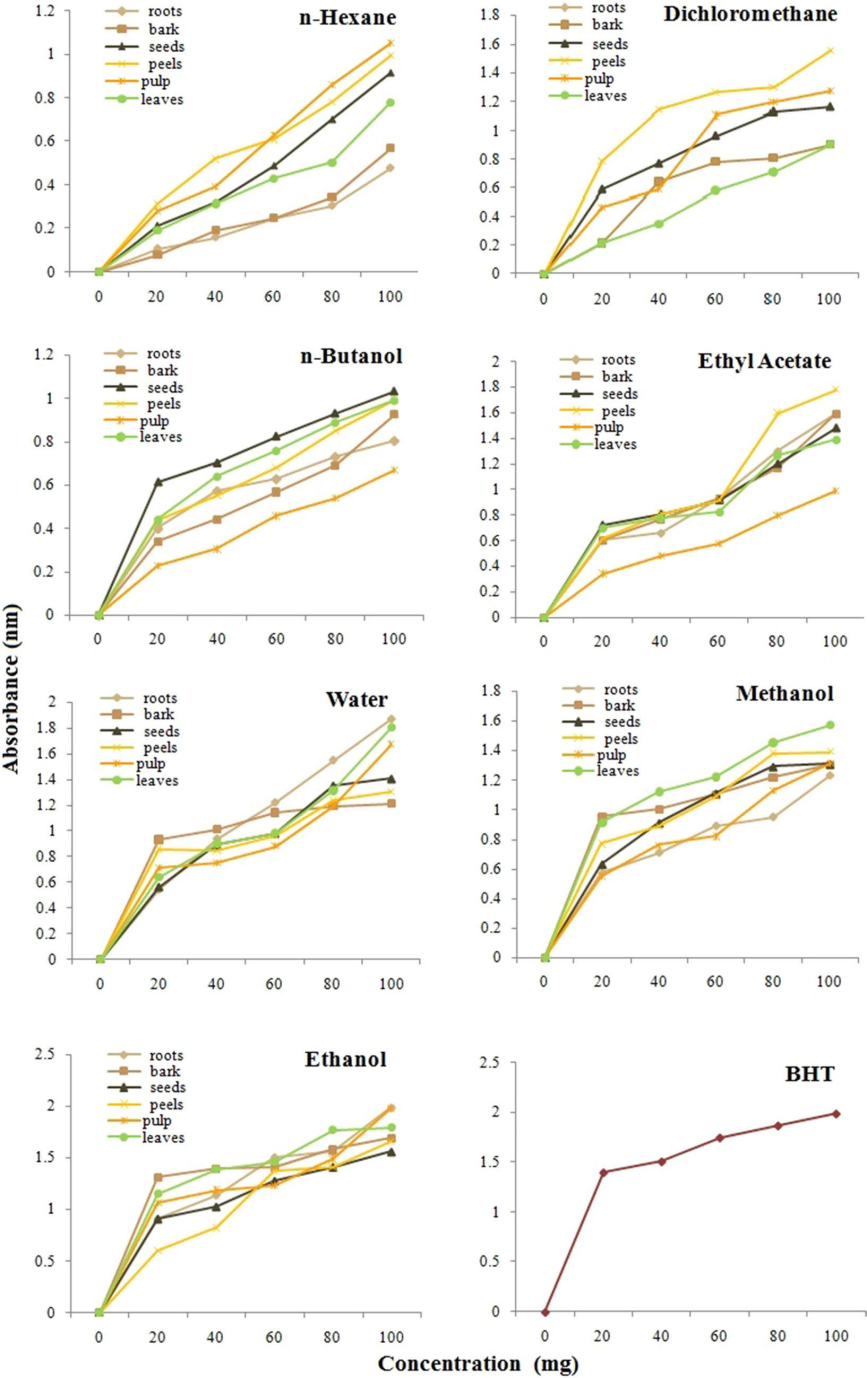
Reducing power potential study of all major parts of *C. papaya* extracts in aqueous and organic solvents

Figure 4 shows the reducing power of different parts of *C. papaya* as a function of concentration. The assay bases on the gradual color change by reduction of the oxidants as function of reducing agent concentration. In this assay, the yellow color of the test solution appears due to the Fe^3+^/ferricyanide complex which gradually changes to different shades of green and blue colors on gradual reduction of Fe^3+^–Fe^2+^ as concentration of antioxidant increases. The reduced Fe^3+^–Fe^2+^ concentration is determined by measuring the absorption of Perl’s Prussian blue at 700 nm [29]. The absorption is directly related to reducing power and consequently antioxidant potential. The highest reducing power was found with ethanol solvent extract (absorbance 1.99) followed by water (absorption 1.87) and methanol (absorption 1.57). The least absorbance was observed with n-hexane solvent extract of roots (absorbance 0.48). Other extracts such as n-butanol, dichloromethane and ethyl acetate extracts showed absorbance in the range of 0.6–1.2. While in contrast to all extracts, BHT which is taken as control showed absorbance 1.99 at 100 μg/mL concentration which is comparable to ethanol extract of root (absorbance 1.99) and pulp (absorbance 1.98).

### Antibacterial activity

Antibacterial activities of different part extracts of *C. papaya* against multidrug resistance bacterial strains were listed in Table 5. Statistical analysis showed non-significant to significant difference (P ≤ 0.05, P ≤ 0.01 or P ≤ 0.001). Organic and aqueous solvent extracts were tested against four bacterial strains i.e. *Staphylococcus aureus,* and *Bacillus cereus*(Gram-positive)*, Escherichia coli* and *Pasteurellamultocida*(Gram-negative). The antibacterial activity result showed the ethanolic extract of pulp was more active against bacterial strains (zone of inhibition 16–18 mm) as compared to other solvent extracts. Ethanolic extract of leaves also showed the zoon of inhibition in the range of 14–16 mm against all four bacterial strains, while the minimum zone of inhibition was found in the case of n-hexane extract of roots (3.8 mm). Ethyl acetate, n-butanol, dichloromethane and water extracts of different parts was remained limited to 10 mm zone of inhibition while ethyl acetate and dichloromethane extracts of pulp showed zone of inhibition up to 12 mm. The low bacterial growth inhibition might be due the absence of structural interaction between solvent, extracted compounds and bacterial strains. This has been evidenced in literature that the compounds of same class behave differently with bacterial strains such as quinolone based antibiotics encounter bacteria action with different efficacy and also face different mode of resistance from bacterial strains as well. Similarly different phenolic compounds and other biological active compounds extracted from natural sources also behave differently in different biological systems. Different solvents don’t extract similar kind of natural compounds with same concentration and consequently don’t show biological activities with same potential. Ethanolic extracts followed by methanolic extracts only presented the best antibacterial activity against both gram positive and gram negative tested bacterial strains due to its great ability to extract those polyphenolic and biological active compounds from natural sources which effectively act against broad spectrum bacteria. The weak antibacterial potential of water extracts is in good agreement with literature reports that hydrophobicity often act as domain driver of antibacterial activities [30, 31].

**Table 5:** Antibacterial activity of all major part extracts in aqueous and organic solvent of *C. papaya* against gram positive and gram negative bacterial strains (mean ± SE; *n* = 3)

Organisms	n-hexane	Dichloro-methan	n-butanol	Ethyl-acetate	Water	Methanol	Ethanol	Cipro-floxacin
Pulp								
*S.aureus*	7.8 ± 0.2^b^	10.5 ± 0.5^ab^	9.0 ± 0.5^b^	11.9 ± 1.3^a^	9.0 ± 0.5^a^	13.0 ± 0.0^b^	17.8 ± 0.0^a^	21.5 ± 0.8
*B. cereus*	6.8 ± 0.5^c^	10.1 ± 0.5^ab^	10.0 ± 0.2^a^	12.2 ± 0.6^a^	8.7 ± 0.8^a^	12.9 ± 0.8^b^	15.9 ± 0.8^a^	18.6 ± 0.6
*E. coli*	9.3 ± 0.1^a^	11.2 ± 0.1^a^	9.0 ± 0.1^b^	10.1 ± 0.0^a^	9.2 ± 0.0^a^	11.5 ± 0.1^b^	16.9 ± 0.0^a^	22.3 ± 1.1
*P. multocida*	7.9 ± 0.3^b^	9.7 ± 0.7^b^	10.0 ± 0.9^a^	11.5 ± 1.2^a^	7.9 ± 2.4^a^	14.8 ± 1.7^a^	18.1 ± 1.2^a^	21.2 ± 1.2
LSD 5 %	0.59***	0.94*	1.01*	1.76 ^ns^	2.41 ^ns^	1.80*	1.40*	
Leaves								
*S.aureus*	6.7 ± 0.2^a^	6.7 ± 0.2^b^	5.9 ± 0.4^c^	9.2 ± 0.3^a^	7.3 ± 0.1^c^	9.2 ± 0.2^d^	16.2 ± 0.3^a^	21.5 ± 0.8
*B. cereus*	5.7 ± 0.2^b^	5.2 ± 0.4^d^	5.9 ± 0.4^c^	7.2 ± 0.3^c^	9.0 ± 0.2^b^	11.0 ± 0.1^b^	14.5 ± 0.2^c^	18.6 ± 0.6
*E. coli*	6.9 ± 0.0^a^	6.3 ± 0.0^c^	7.5 ± 0.1^b^	8.2 ± 0.2^b^	9.0 ± 0.1^b^	10.0 ± 0.4^c^	14.3 ± 0.1^c^	22.3 ± 1.1
*P. multocida*	5.6 ± 0.9^b^	7.2 ± 0.2^a^	8.1 ± 0.1^a^	9.2 ± 0.2^a^	9.2 ± 0.1^a^	13.2 ± 0.5^a^	15.3 ± 0.3^a^	21.2 ± 1.2
LSD 5 %	0.97*	0.31***	0.60***	0.45***	0.25***	0.62***	0.40***	
Seed								
*S.aureus*	5.5 ± 0.2^b^	4.6 ± 0.0^c^	9.0 ± 0.2^b^	9.7 ± 0.4^b^	8.7 ± 0.3^c^	9.9 ± 0.4^b^	14.0 ± 0.3^a^	21.5 ± 0.8
*B. cereus*	6.1 ± 0.3^a^	7.3 ± 0.4^a^	9.5 ± 0.1^a^	9.1 ± 0.1^c^	10.1 ± 0.0^a^	11.3 ± 0.0^a^	11.7 ± 0.2^b^	18.6 ± 0.6
*E. coli*	5.0 ± 0.1^c^	6.8 ± 0.0^b^	8.7 ± 0.2^c^	10.5 ± 0.0^a^	9.6 ± 0.1^b^	10.9 ± 0.3^a^	14.0 ± 0.1^a^	22.3 ± 1.1
*P. multocida*	6.3 ± 0.2^a^	6.5 ± 0.3^b^	8.4 ± 0.2^c^	9.0 ± 0.1^c^	10.4 ± 0.3^a^	10.1 ± 0.1^b^	13.8 ± 0.1^a^	21.2 ± 1.2
LSD 5 %	0.36***	0.47***	0.35***	0.38***	0.38***	0.49***	0.31***	
Roots								
*S. aureus*	4.0 ± 0.1^b^	7.0 ± 1.0^a^	8.0 ± 0.3^a^	8.3 ± 0.3^b^	7.2 ± 0.4^a^	10.2 ± 0.4^a^	10.5 ± 1.0^a^	21.5 ± 0.8
*B. cereus*	3.8 ± 0.9^b^	7.0 ± 0.9^a^	7.6 ± 0.6^a^	8.2 ± 0.6^b^	8.0 ± 0.4^a^	9.3 ± 0.2^a^	9.5 ± 0.0^a^	18.6 ± 0.6
*E. coli*	5.8 ± 0.0^a^	8.6 ± 0.1^a^	9.1 ± 0.0^a^	8.0 ± 0.4^b^	8.5 ± 0.4^a^	9.9 ± 0.2^a^	11.0 ± 0.0^a^	22.3 ± 1.1
P. multocida	5.5 ± 0.1^a^	8.1 ± 0.1^a^	8.7 ± 1.0^a^	9.0 ± 0.0^a^	10.0 ± 1.9^a^	10.2 ± 1.2^a^	11.7 ± 1.0^a^	21.2 ± 1.2
LSD 5 %	0.86**	1.24*	1.11 ^ns^	0.61*	1.89*	1.26 ^ns^	1.31*	
Peels								
*S. aureus*	5.4 ± 0.1^a^	5.6 ± 0.0^b^	6.4 ± 0.1^d^	7.0 ± 0.4^a^	7.7 ± 0.4^a^	8.1 ± 0.3^c^	12.5 ± 0.3^c^	21.5 ± 0.8
*B. cereus*	5.3 ± 1.5^a^	5.6 ± 0.2^b^	6.7 ± 0.0^c^	7.4 ± 0.1^ab^	7.9 ± 0.9^a^	8.12 ± 0.1^c^	14.8 ± 0.2^a^	18.6 ± 0.6
*E. coli*	6.5 ± 0.0^a^	5.8 ± 0.1^ab^	7.3 ± 0.1^a^	8.0 ± 0.4^a^	8.6 ± 0.2^a^	9.1 ± 0.0^a^	13.6 ± 0.3^b^	22.3 ± 1.1
*P. multocida*	5.9 ± 1.3^a^	6.0 ± 0.1^a^	7.0 ± 0.2^b^	7.8 ± 0.3^a^	8.0 ± 0.0^a^	8.8 ± 0.1^b^	12.5 ± 1.2^c^	21.2 ± 1.2
LSD 5 %	1.87 ^ns^	0.26*	0.18***	0.62*	0.96 ^ns^	0.13***	0.83*	
Bark								
*S.aureus*	6.9 ± 0.0^a^	7.1 ± 1.2^a^	7.5 ± 1.2^a^	8.0 ± 1.9^a^	8.0 ± 1.1^a^	8.9 ± 1.3^a^	10.9 ± 1.1^a^	21.5 ± 0.8
*B. cereus*	6.1 ± 0.0^b^	7.3 ± 1.0^a^	7.4 ± 1.5^a^	8.5 ± 0.3^a^	8.0 ± 0.1^a^	9.1 ± 1.5^a^	10.5 ± 1.6^a^	18.6 ± 0.6
*E. coli*	5.9 ± 0.1^b^	7.8 ± 0.0^a^	8.0 ± 0.1^a^	8.9 ± 0.2^a^	9.0 ± 0.0^a^	10.5 ± 0.0^a^	11.0 ± 0.0^a^	22.3 ± 1.1
*P. multocida*	7.5 ± 0.7^a^	7.6 ± 0.3^a^	8.0 ± 1.2^a^	9.4 ± 1.4^a^	8.3 ± 0.7^a^	10.0 ± 2.1^a^	11.0 ± 1.0^a^	21.2 ± 1.2
LSD 5 %	0.66**	1.50 ^ns^	2.16 ^ns^	2.23 ^ns^	1.22 ^ns^	2.72 ^ns^	2.06 ^ns^	

Values with same letter in superscript in row do not differ significantly

*NS* non-significant

***, ** and * significant at 0.001, 0.01 and 0.05 levels respectively

Antibacterial study was performed using ciprofloxacin as a control antibacterial agent. It appears slightly more efficient to stop bacterial growth as compared to the highly active ethanolic pulp extract.

## Methods

### Plant materials

Generally different parts of the plants exhibit chemical composition varying from each other according to the cultivar conditions [32]. *C. papaya* fruit, leaves, bark and roots were collected from selected harvested areas of the lower Punjab province of Pakistan and used throughout this study. All parts of *C. papaya* were washed gently with tape water and then by using distilled water followed by drying (under shade) and grinding. The freeze drying method was also used to dry peel and pulp.

### Trace element analysis

For the preparation of samples to analyze mineral composition, wet digestion procedure was used. Briefly, to 1 g of sample in a beaker added 5 mL of conc. HNO_3_. The solution was boiled till the volume was reduced to 1 mL. Then 2 mL Hydrogen Peroxide (H_2_O_2_) was added drop wise till the solution become clear followed by the dilution with 25 mL of deionized water. Trace and heavy elements in the samples of leaf, peel and pulp were analyzed by the use of Atomic Absorption Spectrophotometer (Hitachi Polarized Zeeman AAS, Z-8200, Japan) following the conditions described in AOAC (1990). Biological active metals included Cobalt (Co), Copper (Cu), Lead (Pb), Iron (Fe) and Zinc (Zn) were selected to measure.

### Preparation of extracts

Dried and stored powdered sample was extracted with each of the following solvents; methanol, ethanol, ethyl acetate, dichloromethane, n-butanol and n-hexane, in a 1:10 (w/v) ratio of *C. papaya* part to solvent, for 2 weeks with periodic shaking at regular intervals. After the extraction, the contents were filtered through Whatman # 1 filter paper, followed by centrifugation at 13000 g for 5 min. Then all the filtrates were evaporated at room temperature or with rotary evaporator in case of more polar solvent system. The dry extract was then used to calculate the percent yield and further analysis.

### Determination of total phenolic contents

Total phenolics in selected part of *C. papaya* were determined using the Folin–Ciocalteau reagent method [33]. Briefly, to the 50 mg extract added 0.5 mL Folin-Ciocalteu reagent which was then diluted with 7.5 mL deionized water. The solution was then shaked well and kept it at room temperature for 10 min followed by the addition of 1.5 mL sodium carbonate (Na_2_CO_3_) solution (20 %) and heated at 40 °C for 20 min in water bath. The heated solution was then cooled in ice bath and took absorbance at 755 nm. The results were then compared with calibrated gallic acid curve and finally results were represented as mg gallic acid equivalent (GAE) per g dry matter.

### Determination of total flavonoid contents

Total flavonoids were analyzed by commonly adopted procedure described by Dewanto et al. [34]. Briefly, to 1 mL of the test solution (0.1 g/mL) added 5 mL distilled water followed by following steps; addition of 0.3 mL of 5 % Sodium Nitrite, incubation for 5 min, addition of 0.6 mL of 10 % AlCl_3_, and addition of 2 mL Sodium Hydroxide (1 M) after another 5 min incubation period. The whole mixture was then diluted to 10 mL by adding distilled water. The mixture was then well shaked and took absorbance at 510 nm. Total flavonoids were calculated in mg CE per g dry matter.

### Determination of antioxidant activity

Antioxidant activity of different extracts of various parts of papaya was assessed by three different assays namely reducing power, inhibition of linoleic acid peroxidation assay and DPPH free radical scavenging activity.

### Determination of reducing power

The reducing power of various parts of *C. papaya* was determined using procedure described by Yen & Duh with slight modification [35]. Different extracts of 2–10 mg was added to 5 mL of sodium phosphate buffer (pH 6.6) followed by the addition of 5 mL potassium ferricyanide (1 %) and the mixture was heated at 50 °C for 20 min. After heating step, 5 mL trichloroacetic acid (10 %) was added and centrifuged the mixture at 1000 rpm for 10 min at 4 °C. To the first layer of centrifuged mixture added 5 mL distilled water and 1 mL ferric chloride solution (0.1 %). Absorbance of the solution was determined at 700 nm. All the samples were analyzed thrice and the average of the results was taken.

### Determination of DPPH free radical scavenging potential

DPPH free radical scavenging activity of different extracts of *C. papaya* was determined by following the method described by Iqbal et al. [36]. According to the procedure, to 1 mL of ethanolic extract solution (25 µg/mL), added 5 mL methanolic solution of 2,2′-diphenyl-1-picrylhydrazyl (DPPH) solution of 0.025 g/L concentration. The contents were vortexed for 1 min and left to stand at room temperature for 20 min followed by measuring the absorbance at 510 nm. Free radical scavenging activity was calculated using the following formula.

$${\text{DPPH Inhibition }}\left( \% \right) \; { = }\;\left[ { 1 {{-A}}_{ 1} / {\text{A}}_{ 0} } \right] { \times \, } 100 {\text{ (A}}_{1} {\text{ = Absorbance of sample, A}}_{0} {\text{ = Absorbance of control)}}$$

The assay was replicated thrice for each sample and result was taken as mean ± standard deviation.

### % Inhibition of linoleic acid peroxidation

Antioxidant activity of all extract was determined using linoleic acid model system reported previously [37]. Briefly, in different extracts of *C. papaya* containing 5 mg of dry extract added 0.13 mL of linoleic acid, 10 mL of pure ethanol, 10 mL Sodium phosphate buffer (0.2 M, pH 7). The total volume of the mixture was made up to 25 mL with distilled water and the mixture was incubated for 172 h at 37 °C. At the end of 172 h the linoleic acid peroxidation inhibition was determined by thiocyanate method. Briefly, to 10 mL of 75 % ethanol added 0.2 mL of sample solution, 0.2 mL of ferrous chloride solution (FeCl_2_) (20 mM in 3.5 % HCl) and mixed sequentially. The solution was then stirred for 3 min and absorbance was noted at 500 nm. A solution with linoleic acid but without sample was taken as negative control and solution containing synthetic standard antioxidant, BHT was taken as positive control.

### Antibacterial activity

Antibacterial activity of different *C. papaya* extracts were measured using disc diffusion method as described earlier with slight modification [38]. Antibacterial activity was assessed against four bacterial strains *S. aureus, E. coli, B. cereus, and P. multocida*. Twenty milliliter media containing bacterial strain was poured into nutrient agar petri plats and allowed to set. After that, sterile filter paper discs (10 mm) placed on surface of the medium followed by loading 100 μL sample (10 mg/ml) dissolved in DMSO onto filter discs. The solution of same concentration of ciprofloxacin was also loaded as positive control. Petri plates were then incubated for 18–24 h at 37 °C in an incubator. At the end of incubation period zone of inhibitions was measured by zone reader.

### Statistical analysis

The experiment were designed in a completely randomized design (CRD) with three replicates and data so generated for different attributes was analysed using a software named CoSTAT V 6.3 (developed by, Cohort software, Berkeley, California, USA).

## Conclusion

This study was conducted to test the medicinal profile of all parts of *C. papaya* by extracting secondary metabolites with organic and aqueous solvents. Secondary metabolites are associated with numerous biological processes in living body, for example; defense system, biotic and abiotic stress. Total 42 extracts of different parts of *C. papaya* were examined using key in vitro biological assay models. Methanol and ethanol extracts of roots and bark showed good antioxidant activities in addition to leaves, peel and pulp extracts; however, methanol and ethanol extract of pulp and leaves showed promising antibacterial activities in addition to antioxidant potential. Ethanol and methanol both were found to be the best solvents of choice to extract natural products to get maximum medicinal benefits. The results obtained from this study could be more beneficient if individual or combined extraction of pulp, leaves, bark or peels is carried out with ethanol for preparing ready to use extracts to combat oxidative stress and bacterial infections.

## Abbreviations

*C. Papaya*: *Carica papaya*
*S. aureus*: *Staphylococcus aureus*
*E. coli*: *Escherichia coli*
*B. cereus*: *Bacillus cereus*
*P. multocida*: *Pasteurellamultocida*
TPC: total Phenolic Contents
TFC: total Flavonoid Contents
DPPH: 2,2-diphenyl-1-picrylhydrazyl
ROS: reactive oxygen species
RNS: reactive nitrogen species

## References

[1] Dominique B, Sylvie L, Simon LD, Jessica J, Edith B, Martine C (2009). Antiproliferative and antioxidant activities of common vegetables vegetables; a comparative study. Food Chem.

[2] Miyake Y, Fukushima W, Tanaka K, Sasaki S, Kiyohara C, Tsuboi Y (2011). Dietary intake of antioxidant vitamins and risk of Parkinson’s disease: a case-control study in Japan. Eur J Neurol.

[3] Zeynep S. Agim and Jason R (2015) Cannon “dietary factors in the etiology of parkinson’s disease”. Bio Med Res. Article ID 672838, 16 pages. doi:10.1155/2015/672838 10.1155/2015/672838 PMC4320877 25688361

[4] SchwabU Lauritzen L, Tholstrup T, Haldorsson TI, Riserus U, Uusitupa M, Becker W (2014). Effect of the amount and type of dietary fat on cardiometabolic risk factors and risk of developing type 2 diabetes, cardiovascular diseases, and cancer: a systematic review. Food Nut Res.

[5] Uttara B, Singh AV, Zamboni P, Mahajan R (2009). Oxidative stress and neurodegenerative diseases: a review of upstream and downstream antioxidant therapeutic options. Curr Neuropharmacol.

[6] Boskou D (2006). Sources of natural phenolic antioxidants. Trends Food Sci Technol.

[7] Yan Z, Baolu Z (2013). Oxidative stress and the pathogenesis of alzheimer’s disease. Oxid Med Cell Longev.

[8] Madeo J, Elsayad C (2013). The Role of oxidative stress in alzheimer’s disease. J Alzheimers Dis Parkinsonism.

[9] Tsang AH, Chung KK (2009). Oxidative and nitrosative stress in Parkinson’s disease. Biochim Biophys Acta.

[10] Maria MB, Almeida Paulo HMS, Ângela MCA, Giovana MP, Carlos Emanuel CM, Geraldo AM (2011). Bioactive compounds and antioxidant activity of fresh exotic fruits from northeastern Brazil. Food Res In.

[11] Marisa MW (2006). Ascorbic acid, vitamin A, and mineral composition of banana (Musa sp.) and papaya (*Carica papaya*) cultivars grown in Hawaii. J Food Compost Anal.

[12] Otsuki N, Dang NH, Kumagai E, Kondo A, Iwata S, Morimoto C (2010). Aqueous extract of *Carica papaya* leaves exhibits anti-tumor activity and immunomodulatory effects. J Ethnopharmacol.

[13] Nguyen Thao TTS, Paul NP, Marie O, Hewa V, Amitha K (2013). Anticancer activity of *Carica papaya*. Mol Nutr Food Res J.

[14] Metrouh-Amir H, Duarte CMM, Maiza F (2015). Solvent effect on total phenolic contents, antioxidant, and antibacterial activities of Matricaria pubescens. Ind Crop Prod.

[15] Hauppauge Nabrzyski M (2013). Functional role of some minerals in foods. Miner Compon Food.

[16] Grembecka M, Szefer P (2013). Comparative assessment of essential and heavy metals in fruits from different geographical origins. Environ Monit Assess.

[17] Zakia K, Kong HS, NurHazerra BMZ, Chua HC, Irshad UHB (2015). Determination of polyphenolic content, HPLC analyses and DNA cleavage activity of Malaysian Averrhoacarambola L. fruit extracts. J King Saud Univ Sci.

[18] Chirinos R, Rogez H, Campos D, Pedreschi R, Larondelle Y (2007). Optimization of extraction conditions of antioxidant phenolic compounds from mashua (Tropaeolum tuberosum Ruiz and Pavon) tubers. Sep Purif Technol.

[19] Turkmen N, Sar F, VeliÔglu YS (2006). Effects of extraction solvents on concentration and antioxidant activity of black and black mate tea polyphenols determined by ferrous tartrate and Folin-Ciocalteu methods. Food Chem.

[20] Witayapan N, Songwut YW, Siriporn O (2010). Factors influencing antioxidant activities and total phenolic content of guava leaf extract. LWT-Food Sci Technol.

[21] Quan VV, Sathira H, Paul DR, Michael CB, Phoebe AP, Christopher JS (2013). Effect of extraction conditions on total phenolic compounds and antioxidant activities of *Carica papaya* leaf aqueous extracts. J Herb Med.

[22] Lee SC, Norliza AL, SzeYL ChewTL, Mohamad RS, Ramlan AA (2011). Flavonoids and phenolic acids from *Labisiapumila* (*Kacip Fatimah*). Food Chem.

[23] Ock KC, Sang JC, Won OS (2007). Estimated dietary flavonoid intake and major food sources of US. Adults J Nutr.

[24] Shashank K, Abhay K.P (2013) Chemistry and biological activities of flavonoids, an overview. Sci World J Article ID 162750, 16 pages .http://dx.doi.org/10.1155/2013/162750 10.1155/2013/162750 PMC3891543 24470791

[25] James MH, Zobert FD, Zary RB, Joanne MH, David BH, Seema B (2006). Flavonoid Content of US Fruits, Vegetables, and Nuts. Food Chem.

[26] Joana SB, Érica OB, Beatriz CS, Paula FM, Vitor CA, Jesuí VV (2014). Evaluation of solvent effect on the extraction of phenolic compounds and antioxidant capacities from the berries application of principal component analysis. Chem Cent J.

[27] Amarowicz R, Pegg RB, Rahimi PM, Barl B, Weil JA (2004). Free-radical scavenging capacity and antioxidant activity of selected plant species from the Canadian prairies. Food Chem.

[28] Isabel CFR, Ferreira Paula B, Miguel VB, Lillian B (2007). Free-radical scavenging capacity and reducing power of wild edible mushrooms from northeast Portugal individual cap and stipe activity. Food Chem.

[29] Rahmat AK (2012). Evaluation of flavonoids and diverse antioxidant activities of Sonchusarvensis. Chem Cent J.

[30] Dean GB, Tricia LMD, Moriah MG, Ruben T (2014). Trends and exceptions of physical properties on antibacterial activity for gram-positive and gram-negative pathogens. J Med Chem.

[31] Iyer R, Ferrari A, Rijnbrand R, Erwin AL (2015). A fluorescent microplate assay quantifies bacterial efflux and demonstrates two distinct compound binding sites in AcrB. Antimicrob Agents Chem.

[32] Witayapan Nantitanon, Songwut YW, Siriporn O (2010). Factors influencing antioxidant activities and total phenolic content of guava leaf extract. Food Sci Technol.

[33] Sultana B, Anwar F, Ashraf M, Saari N (2012). Effect of drying techniques on the total phenolic contents and antioxidant activity of selected fruits. J Med Plants Res.

[34] Dewanto v, Wu x, Adom KK, Liu RH (2002). Thermal processing enhances the nutritional value of tomatoes by increasing total antioxidant activity. J Agric Food Chem.

[35] Yen GC, Duh PD (1994). Scavenging effect of methanolic extracts of peanut hulls on free radical and active oxygen species. J Agric Food Chem.

[36] Iqbal S, Bhanger MI, Anwer F (2005). Antioxidant properties and components of some commercially available varieties of rice bran in Pakistan. Food Chem.

[37] Naqvi SAR, Mahmood N, Naz S, Hussain Z, Sherazi TA, Khan ZA (2013). Antioxidant and antibacterial evaluation of honey bee hive extracts using in vitro models. Mediterr J Nutr Metab.

[38] Sahar A, Naqvi SAR, Hussain Z, Nosheen S, Khan ZA, Ahmad M (2013). Screening of phytoconstituents, investigation of antioxidant and antibacterial activity of methanolic and aqueous extracts of Cucumissativus. J Chem Soc Pak.

